# Revisiting time-dependent growth and nucleation rates in the Johnson–Mehl–Avrami–Kolmogorov equation

**DOI:** 10.1098/rsos.241696

**Published:** 2025-05-14

**Authors:** Kiana Shirzad, Christopher Viney

**Affiliations:** ^1^ Graduate Program in Materials and Biomaterials Science and Engineering, University of California Merced, Merced, CA, USA; ^2^ Department of Chemical and Materials Engineering, University of California Merced, Merced, CA, USA

**Keywords:** diffusion, interface control, Johnson–Mehl–Avrami–Kolmogorov (JMAK) equation, nucleation and growth, phase transformation, reaction equation

## Abstract

The Johnson–Mehl–Avrami–Kolmogorov (JMAK) equation is widely used to model phase transformation kinetics—a topic central to materials in which solidification, precipitation or recrystallization is occurring. The classic derivation of this reaction equation primarily assumes that nucleation is either instantaneous or sporadic at a constant rate, and that growth rate is constant; consideration of possible time dependence of these rates is limited to the physically uncommon cases of the time dependence being linear. In contrast, many common phase transformations involve kinetics in which the growth rate varies in proportion to time raised to a power that can range from −0.5 (diffusion control; Fickian diffusion; case I diffusion), through values that represent anomalous diffusion, to zero (interface control; case II diffusion) and beyond (super case II diffusion). We have extended the classic derivation by generalizing the formulation of the growth and nucleation rates to include these contexts, and we introduce and justify some additional refinements while retaining the overall mathematical accessibility of the classic derivation. The reaction equations derived in this process explicitly demonstrate how the time dependence of nucleation rate and growth rate affects the constants in the JMAK equation, allowing the possibility of values beyond their classical range.

## 1. Introduction

The study of phase transformations holds great importance in chemistry and materials science. It provides a tool for the control of microstructure and therefore the tuning of material properties [1]. There have been numerous efforts to describe and predict the kinetics of phase transformations; many have resulted in the development of sophisticated and comprehensive models [2–6]. However, in many cases, simpler models that speak to a broader audience and provide helpful insights in an accessible language can be preferrable. The Johnson–Mehl–Avrami–Kolmogorov (JMAK) formalization, commonly referred to as the Avrami equation, has found widespread use since its origins in the late 1930s and early 1940s. It was developed independently by Johnson & Mehl [7], Avrami [8–10] and Kolmogorov [11,12], and describes the progress of nucleation-and-growth-type phase transformations as a simple sigmoidal function [13]:


(1.1) $$f\left(t\right)=1-\exp\left(-kt^{n}\right),$$

where f(t) is the transformed fraction of material as a function of time. The Avrami coefficient (rate constant) *k* is a function of both nucleation rate and growth rate, and depends on the geometry of the growing nuclei; the Avrami exponent n depends on the dimensionality of growth and on whether nucleation is instantaneous or sporadic [14].

The JMAK equation offers a simple model with minimal computational burden and has been applied to various transformations within and beyond materials science [15,16]. The equation is typically used under the assumption that nucleation is either instantaneous (restricted to a very short time interval at the start of the transformation) or sporadic and independent of time. Growth rate is also assumed to be independent of time. In contrast, many real processes involve a time-dependent growth rate, which may reflect a time dependence of concentration gradients and resultant diffusion rates. For example, for transformations that are under diffusion control, when a solute has to be brought to the growth interface from increasing distances and therefore under a declining concentration gradient, the growth is proportional to $t^{1/2}$ and the growth *rate* is therefore proportional to $t^{-1/2}$. Diffusion control is accommodated in the Avrami exponent n by allowing it to assume half-integral values [17,18], but the time dependence of growth rate is ignored in the Avrami coefficient k.

In their landmark paper, Johnson & Mehl consider growth rates and nucleation rates that vary *linearly* with time. Their paper was published [7] in print form without its appendices. More recently, the entire paper, including all five appendices A–E, has been republished, along with a related discussion of its historical context and significance [19,20]. In the current work, we build on the foundation provided by Johnson & Mehl, with the goal of enhancing the versatility of the JMAK model and broadening its applicability. We derive a reaction equation that incorporates possible time dependences of both growth rate and nucleation rate under conditions of Fickian diffusion, anomalous diffusion, case II diffusion, and super case II diffusion, explicitly stating the effect on both n and k. (Refer to Langer & Peppas [21] for a description of the abovementioned diffusion types.)

We emphasize from the outset that the original JMAK model and our extension thereof are not formulated in terms of the autonomous differential equations of chemical kinetics. Situations in materials chemistry where the latter approach can be rigorously applied include the formation of a product that is readily described in terms of the explicit attachment of additional monomer units to growing aggregates, as in the case of nanoparticle growth [22]. However, in many of the phase transformations encountered in materials chemistry, the parent and/or product phases are random solid solutions (with a statistical distribution of the component atom types on the crystal lattice), and each phase may be stable over a finite range of compositions at a given temperature. Measurement of time- and location-dependent compositional changes is challenging. Practically, the interest lies in modelling and predicting the amount of transformed material as a function of time—and not in calculating the underlying concentration profiles. Therefore, the JMAK model does not start with expressions of time-dependent concentration, but with the practically more accessible nucleation and growth rates that are consequences of the concentration changes. We also note that the transformations encountered in materials chemistry often occur over extended time periods (minutes, hours or even days) in comparison to nanoparticle formation, and it is the former that are of particular interest to us.

Our reaction equation is developed in §2, in a series of scenarios. We start (scenario 1) with Johnson & Mehl’s derivation [7] in which growth rate G(t) and nucleation rate N(t) are linear functions of time, and we provide the mathematical steps that are omitted in Johnson & Mehl’s account of the derivation. Then (scenario 2) we propose and justify a refinement to how the linear time dependences of G(t) and N(t) are represented. Next (scenario 3), we generalize the formulation of G(t) and N(t) to include several practically relevant cases of nonlinear time dependence, with a focus on growth. In scenario 4, we return to Johnson & Mehl’s derivation to propose and discuss a modification to the integration limits used for calculating growth. In scenario 5, we combine the refinements that were introduced in scenarios 4 and 2. Finally, in scenario 6, we combine the refinements that were introduced in scenarios 4 and 3.

Section 3 focuses on the time dependence of nucleation rate, along with cases of instantaneous nucleation where a nucleation rate is not defined. Situations in which the Avrami exponent *n* can exceed the classical maximum value of 4 are identified.

Section 4 provides comparisons between predictions made by the different scenarios, and highlights the capacity of our generalized reaction equation to accommodate a range of standard diffusion types.

## 2. The reaction equation for cases in which growth rate and nucleation rate vary with time

### 2.1. Scenario 1: Johnson & Mehl’s derivation of the reaction equation for cases where growth rate and nucleation rate vary *linearly* with time

The derivation is re-created below; the mathematical detail is similar to that presented by Johnson & Mehl [7]. A comprehensive version, with all steps explicitly shown, is provided as electronic supplementary material, S1.

Growth rate (the rate at which size r changes in one dimension) varies linearly with time according to


(2.1) $$\frac{dr}{dt}=G\left(t\right)=G_{0}+G_{0}\alpha t=G_{0}\left(1+\alpha t\right),$$

where $G_{0}$ and α are constants.

Nucleation rate varies linearly with time according to


(2.2) $$\begin{array}{r} \frac{dn}{dt}=N\left(t\right)=N_{0}+N_{0}\beta t=N_{0}\left(1+\beta t\right), \end{array}$$

where $N_{0}$ and β are constants.

At time t, the radius of a sphere that nucleated at time T<t is


(2.3) $$r=G_{0}\int_{T}^{t}\left(1+\alpha t\right)dt=\frac{G_{0}}{2\alpha }\;\left[{\left(1+\alpha t\right)}^{2}-{\left(1+\alpha T\right)}^{2}\right].$$

The volume of a sphere is


$$\phi_{1}=\frac{4}{3}\pi r^{3}.$$

The rate of change of volume therefore is


$$\begin{array}{rl} \frac{d\phi_{1}}{dt} & =4\pi r^{2}\frac{dr}{dt}\\ & =\frac{\pi G_{0}^{3}}{\alpha^{2}}\left(1+\alpha t\right){\left[{\left(1+\alpha t\right)}^{2}-{\left(1+\alpha T\right)}^{2}\right]}^{2}. \end{array}$$

If the fraction of accessible (untransformed) matrix remaining at time t is u(t), the effective rate at which the volume of the sphere changes at that time is given by


$$\begin{array}{rl} \frac{d\phi_{2}}{dt} & =u\left(t\right)\frac{d\phi_{1}}{dt}\\ & =u\left(t\right)\;\frac{\pi G_{0}^{3}}{\alpha^{2}}\left(1+\alpha t\right){\left[{\left(1+\alpha t\right)}^{2}-{\left(1+\alpha T\right)}^{2}\right]}^{2}. \end{array}$$

The number of nuclei formed in a short time interval dT at time T is


$$dn=N_{0}\left(1+\beta T\right)\;dT.$$

Therefore, the total rate of volume increase of all spheres nucleated at time T is


$$\frac{d\phi_{3}}{dt}=u\left(t\right)\;\frac{\pi N_{0}G_{0}^{3}}{\alpha^{2}}\left(1+\alpha t\right)\left(1+\beta T\right)\;{\left[{\left(1+\alpha t\right)}^{2}-{\left(1+\alpha T\right)}^{2}\right]}^{2}\;dT.$$

Therefore, the total rate of volume increase of all spheres nucleated from T=0 to T=t is


$$\begin{array}{r} \frac{d\phi_{4}}{dt}=u\left(t\right)\;\frac{\pi N_{0}G_{0}^{3}}{\alpha^{2}}\left(1+\alpha t\right)\int_{0}^{t}\left(1+\beta T\right){\left[{\left(1+\alpha t\right)}^{2}-{\left(1+\alpha T\right)}^{2}\right]}^{2}\;dT\\ =u\left(t\right)\;\pi N_{0}G_{0}^{3}\;\left(1+\alpha t\right)\left[\frac{4}{3}t^{3}+\frac{5\alpha +\beta }{3}t^{4}+\frac{8\alpha^{2}+7\alpha \beta }{15}t^{5}+\frac{\alpha^{2}\beta }{6}t^{6}\right]. \end{array}$$

Since the transformed fraction at any time is $1-u\left(t\right)$, the transformation rate is −du/dt.

Therefore,


$$\frac{d\phi_{4}}{dt}=-\frac{du}{dt}=u\left(t\right)\;\pi N_{0}G_{0}^{3}\;\left(1+\alpha t\right)\left[\frac{4}{3}t^{3}+\frac{5\alpha +\beta }{3}t^{4}+\frac{8\alpha^{2}+7\alpha \beta }{15}t^{5}+\frac{\alpha^{2}\beta }{6}t^{6}\right].$$

The solution to this differential equation is


$$\ln u\left(t\right)=-\pi N_{0}G_{0}^{3}\;\left\{\frac{1}{3}t^{4}+\frac{9\alpha +\beta }{15}t^{5}+\frac{11\alpha^{2}+4\alpha \beta }{30}t^{6}+\frac{16\alpha^{3}+19\alpha^{2}\beta }{210}t^{7}+\frac{\alpha^{3}\beta }{48}t^{8}\right\}.$$

Finally, we obtain the reaction equation


(2.4) $$f\left(t\right)=1-u\left(t\right)=1-\exp\left\{-\pi N_{0}G_{0}^{3}\;\left[\frac{1}{3}t^{4}+\frac{9\alpha +\beta }{15}t^{5}+\frac{11\alpha^{2}+4\alpha \beta }{30}t^{6}+\frac{16\alpha^{3}+19\alpha^{2}\beta }{210}t^{7}+\frac{\alpha^{3}\beta }{48}t^{8}\right]\right\}$$

for cases where growth rate and nucleation rate vary linearly with time.

As a convenient check for consistency, we can set α=β=0, whereupon we obtain the classic Avrami equation for sporadic nucleation and three-dimensional growth at constant rates, as expected:


(2.5) $$f\left(t\right)=1-\exp\left(-\frac{\pi N_{0}G_{0}^{3}}{3}t^{4}\right).$$

### 2.2. Scenario 2: an improved reaction equation, with refinements to the linear time-dependent growth rate and nucleation rate equations in scenario 1

Equations (2.1) and (2.2), expressing linear time dependence of growth rate and nucleation rate, respectively, are mathematically reasonable but physically suboptimal. The quantity $G_{0}$ appears in both the time-independent term and the time-dependent term in equation (2.1), and the coefficient of the time-dependent term conflates two quantities, $G_{0}$ and α. A similar comment applies to the use of $N_{0}$ and β in equation (2.2).

Alternatively, we could rewrite equation (2.1) as


(2.6) $$\frac{dr}{dt}=G\left(t\right)=G_{0}+\alpha t,$$

where $G_{0}$ and α are constants, and we can rewrite equation (2.2) as


(2.7) $$\frac{dn}{dt}=N\left(t\right)=N_{0}+\beta t,$$

where $N_{0}$ and β are constants. The α in equation (2.6) is numerically equal to $G_{0}$ times the α from equation (2.1), and the β in equation (2.7) is numerically equal to $G_{0}$ times the β from equation (2.2).

These alternative formalizations of the two rate equations have the advantage that they use clearly distinct (i.e. single) coefficients to scale each order of time dependence in the rate equations. Adopting coefficients that are uniquely assigned to each term in a power series would be consistent with the convention used in other physical science and engineering contexts.

Replacing equations (2.1) and (2.2) with equations (2.6) and (2.7) in the derivation of the reaction equation from scenario 1, we obtain


(2.8) $$f\left(t\right)=1-\exp\left\{-\pi \left[\begin{array}{c} \frac{N_{0}G_{0}^{3}}{3}t^{4}+\frac{9N_{0}G_{0}^{2}\alpha +G_{0}^{3}\beta }{15}t^{5}+\frac{11N_{0}G_{0}\alpha^{2}+4G_{0}^{2}\alpha \beta }{30}t^{6}\\ +\frac{16N_{0}\alpha^{3}+19G_{0}\alpha^{2}\beta }{210}t^{7}+\frac{\alpha^{3}\beta }{48}t^{8} \end{array}\right]\right\}$$

for cases where growth rate and nucleation rate vary linearly with time.

The derivation follows the same general procedure as was used for scenario 1, and is provided in detail as electronic supplementary material, S2.

As a convenient check for consistency, we can set α=β=0, whereupon we again obtain the classic Avrami equation (2.5).

### 2.3. Scenario 3: an improved reaction equation, with generalizations of the time-dependent growth rate and nucleation rate equations in scenario 2

The linear time dependence of growth rate and nucleation rate introduced by Johnson & Mehl in their appendix B [7,20] serves to illustrate an approach to developing reaction equations; it is not presented as an analysis of the kinetics of a particular known transformation. Johnson & Mehl quote a study performed by Tammann & Crone [23] on the recrystallization of lead, silver and zinc as evidence that crystal growth rate can change (in this case, decrease) during a nucleation and growth process. However, the time dependence of crystal growth rate according to Tammann & Crone’s data is distinctly nonlinear. We are left to speculate on why Johnson & Mehl chose to derive the reaction equation for the case of a *linear* relationship between G and t.

We can achieve more versatile expressions for the relationship between G and t, and between N and t, by writing heuristic adaptations of equations (2.6) and (2.7):


(2.9) $$\begin{array}{rl} \frac{dr}{dt}=G(t)=G & {\;}_{0}+\alpha t^{p}, \end{array}$$

where p is a real number, and


(2.10) $$\begin{array}{rl} \frac{dn}{dt}=N\left(t\right)=N & {\;}_{0}+\beta t^{q}, \end{array}$$

where q is a real number.

These equations incorporate the case of Johnson & Mehl’s linear time dependence of growth rate and nucleation rate, when p=q=1.

Replacing equations (2.1) and (2.2) with equations (2.9) and (2.10) in the derivation of the reaction equation from scenario 1, we obtain


(2.11) $$f\left(t\right)=1-\exp\left\{-4\pi \;\left[\begin{array}{c} \frac{N_{0}G_{0}^{3}}{12}t^{4}\\ +\frac{N_{0}G_{0}^{2}\alpha \;\left(p^{2}+8p+18\right)}{3\left(p+2\right)\left(p+3\right)\left(p+4\right)}\;t^{p+4}\\ +\frac{2G_{0}^{3}\beta }{\left(q+1\right)\left(q+2\right)\left(q+3\right)\left(q+4\right)}t^{q+4}\\ +\frac{N_{0}G_{0}\alpha^{2}\;\left(2p^{2}+13p+18\right)}{\left(p+2\right)\left(p+3\right)\left(2p+3\right)\left(2p+4\right)}\;t^{2p+4}\\ +\;\frac{2N_{0}\alpha^{3}\;}{\left(p+2\right)\left(2p+3\right)\left(3p+4\right)}\;t^{3p+4}\\ +\frac{2G_{0}^{2}\alpha \beta \;\left(p^{2}+8p+3pq+15q+3q^{2}+18\right)}{\left(q+1\right)\left(q+2\right)\left(q+3\right)\left(p+q+2\right)\left(p+q+3\right)\left(p+q+4\right)}t^{p+q+4}\\ +\frac{2G_{0}\alpha^{2}\beta \;\left(2p^{2}+13p+6pq+15q+3q^{2}+18\right)\;}{\left(q+1\right)\left(q+2\right)\left(p+q+2\right)\left(p+q+3\right)\left(2p+q+3\right)\left(2p+q+4\right)}t^{2p+q+4}\\ +\frac{2\alpha^{3}\beta \;}{\left(q+1\right)\left(p+q+2\right)\left(2p+q+3\right)\left(3p+q+4\right)}t^{3p+q+4} \end{array}\right]\right\}.$$

The derivation follows the same general procedure as was used for scenarios 1 and 2, and is provided in detail as electronic supplementary material, S3.

As a convenient check for consistency, we can set p=q=1, whereupon we obtain equation (2.8) from scenario 2, as expected:


$$\begin{array}{rl} f(t) & =1-\exp\left\{-4\pi \left[\begin{array}{c} \frac{N_{0}G_{0}^{3}}{12}t^{4}+\frac{27N_{0}G_{0}^{2}\alpha }{180}t^{5}+\frac{2G_{0}^{3}\beta }{120}t^{5}+\frac{33N_{0}G_{0}\alpha^{2}}{360}t^{6}\\ +\frac{2N_{0}\alpha^{3}}{105}t^{7}+\frac{96G_{0}^{2}\alpha \beta }{2880}t^{6}+\frac{114G_{0}\alpha^{2}\beta }{5040}t^{7}+\frac{2\alpha^{3}\beta }{384}t^{8} \end{array}\right]\right\}\\ & =1-\exp\left\{-\pi \left[\begin{array}{c} \frac{N_{0}G_{0}^{3}}{3}t^{4}+\frac{9N_{0}G_{0}^{2}\alpha +G_{0}^{3}\beta }{15}t^{5}+\frac{11N_{0}G_{0}\alpha^{2}+4G_{0}^{2}\alpha \beta }{30}t^{6}\\ +\frac{16N_{0}\alpha^{3}+19G_{0}\alpha^{2}\beta }{210}t^{7}+\frac{\alpha^{3}\beta }{48}t^{8} \end{array}\right]\right\}. \end{array}$$

To assess the versatility of equations (2.9) and (2.10) in describing a useful range of real transformations that involve nucleation and growth, we consider them in the context of case I (Fickian) diffusion, case II diffusion (interface control), anomalous diffusion and super case II diffusion. All of these situations are associated with growth that follows an equation of the form


(2.12) $$\begin{array}{rl} r & =r_{0}+Ht^{m}, \end{array}$$

where H is a positive constant, $r_{0}$ is the critical size (linear dimension) for a transformed region to undergo stable growth, and m≥1/2. Table 1 distinguishes between different contexts based on the magnitude of m.

**Table 1.** Different contexts of phase or microstructural transformation, together with the corresponding values of m in equation (2.13) and p in equation (2.9).

case I (Fickian) diffusion; parabolic growth; diffusion control	$m=\frac{1}{2}$	$p=-\frac{1}{2}$
anomalous diffusion	$\frac{1}{2}<m<1$	$-\frac{1}{2}<p<0$
case II diffusion; linear growth; interface control	m=1	p=0
super case II diffusion	m>1	p>0

The growth rate equation corresponding to (2.12) is


(2.13) $$\frac{dr}{dt}=G\left(t\right)=mHt^{m-1}.$$

Equation (2.13) has the form $G\left(t\right)=\alpha t^{p}$, corresponding to equation (2.9) with $G_{0}=0$, α=mH and p=m-1. Values of p are included in table 1.

In principle, values of m down to m=0, and corresponding values of p down to p=-1, are possible; those limits represent situations in which the domains or crystals of the product phase effectively appear at their final size. Still lower values of m and p would imply that the ‘product’ domains or crystals are shrinking.

If m<1 (p<0), i.e. the linear growth rate of spherical nuclei is decelerating, the JMAK approach and our enhancements do not account for some aspects of overgrowth produced by phantom nuclei. The phantom nuclei that form within already-transformed material are addressed by considering only the fraction of remaining (accessible) matrix at each instant of the transformation, as in our derivations. However, in the case of decelerating growth processes, phantom nuclei formed in material that is hitherto untransformed but located close enough to a boundary with already-transformed material can grow beyond that boundary [24]. This aspect of overgrowth has been shown to be almost negligible for growth controlled by Fickian diffusion (m=1/2; p=-1/2), but potentially significant for near-instantaneous growth processes that can occur in nanocrystallization (m∼0; p∼−1) [24]. We shall maintain our focus on cases summarized in table 1 so that, if growth rate deceleration occurs, it is no more severe than the deceleration associated with processes controlled by Fickian diffusion.

One of the main goals of our research is to apply JMAK-based kinetics as an aid to modelling the spread of diseases, such as COVID-19, as justified previously [15]. In that situation, clusters of infection clearly do not appear at final size; the disease spreads through a population over a period of several weeks. The applicability of the results from the present work to descriptively and predictively model the rate of COVID-19 spread, and to identify common features of the spread in different countries, will be addressed in a subsequent publication.

Noting the equivalence between equations (2.13) and (2.9) in the contexts of the transformations collated in table 1, we proceed with equation (2.9) and $G_{0}=0$ in these contexts:


(2.14) $$\frac{dr}{dt}=G\left(t\right)=\alpha t^{p}.$$

Accordingly, we can return to the reaction equation (2.11) and set $G_{0}=0.$ The reaction equation for transformations in contexts where growth kinetics follow case I, case II, anomalous or super case II diffusion kinetics then becomes


(2.15) $$f\left(t\right)=1-\exp\left\{-8\pi \;\left[\begin{array}{c} \;\frac{N_{0}\alpha^{3}\;}{\left(p+2\right)\left(2p+3\right)\left(3p+4\right)}\;t^{3p+4}\\ +\frac{\alpha^{3}\beta \;}{\left(q+1\right)\left(p+q+2\right)\left(2p+q+3\right)\left(3p+q+4\right)}t^{3p+q+4} \end{array}\right]\right\}.$$

If we assume interface control (p=0) and sporadic nucleation at a constant rate $N_{0}$ (β=0), equation (2.15) reduces to the equivalent of equation (2.5) as expected.

Some discrete values of p and q in equations (2.11) and (2.15) are disallowed by the need to ensure that all factors in the denominators of all terms are non-zero. These constraints do not intersect our use of the equations in the stated contexts. Also, we note that an equation such as (2.14) is unbounded, and so would lead to unbounded solutions if used for the purpose of predicting concentration profiles. However, as stated in §1, our practical interest in adopting and extending the JMAK approximation lies in modelling the amount of transformed material as a function of time under various assumed descriptions of nucleation and growth rate. In performing integrations of the assumed nucleation and growth rates, we neglect the existence of a critical radius for nuclei to undergo stable growth instead of redissolving. This simplification (consistent with JMAK theory) is possible because the critical nucleus size typically is small in comparison to the eventual crystal (grain) size. Exceptions will occur in some situations such as those involving nanocrystallization, but, as we have already commented, these lie outside the scope of our modelling.

### 2.4. Scenario 4: an improved reaction equation, modifying the integration limits used for calculating growth in scenario 1

Here, we revisit Johnson & Mehl’s derivation [7,20] of the reaction equation for cases where growth rate and nucleation rate vary linearly with time as presented in scenario 1, retaining their formalizations of the growth rate and nucleation rate equations. We will reconsider the limits of integration that were used when calculating the radius at time t of a sphere that nucleated at time T<t. In scenario 1, the integration is performed as equation (2.3):


$$\begin{array}{rl} r=G_{0}\int_{T}^{t}(1+ & \alpha t)dt=\frac{G_{0}}{2\alpha }\;[(1+\alpha t{)}^{2}-(1+\alpha T{)}^{2}]. \end{array}$$

**Figure 1.**
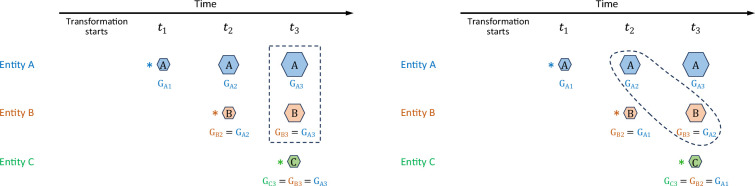
Two models of time-dependent growth rate *G*. Each case schematically follows the growth of three representative entities (e.g. grains, nodules or spherulites) that nucleated at set time intervals after the onset of the transformation. Nucleation, indicated by asterisks, is presumed to occur shortly before the entity is first observed. Broken-line boundaries enclose examples of entities that have the same growth rate. Left: growth rate of each entity depends on time elapsed since onset of transformation (Johnson & Mehl). Right: growth rate of each entity depends on time elapsed since the first appearance of that entity.

Johnson & Mehl’s choice of integration limits [7,20] assumes that the time-dependent growth rate is spatially global within the transforming system: at any given time, each growing entity (e.g. grain, nodule or spherulite) is growing at the same rate as all the other entities, dependent on how long ago the overall transformation started, but regardless of the time elapsed since the particular entity nucleated, and therefore regardless of the particular entity’s current size. This condition is shown schematically in figure 1 (left).

We propose an alternative model that considers the growth rate of any growing entity to depend on the time elapsed since that particular entity nucleated, until its growth is halted by hard impingement. In this consideration of growth rate, the radius at time t of a sphere that nucleated at time T<t should involve a revised integration


(2.16) $$\begin{array}{rl} r & =G_{0}\int_{T-T}^{t-T}(1+at)dt\\ & =G_{0}\int_{0}^{t-T}(1+at)dt\\ & =\frac{\alpha G_{0}}{2}[\frac{2}{\alpha }(t-T)+(t-T{)}^{2}]. \end{array}$$

At any given time, all the growing entities with a particular size are growing at the same rate. This condition is shown schematically in figure 1 (right).

Equation (2.3) in scenario 1 suggests that the chemical environment (source of atomic or molecular ‘building blocks’) surrounding new nuclei is the same as the chemical environment surrounding each of the already established and growing transformed regions. This description in turn suggests either that new nuclei only form at the surface of existing grains (which amounts to a form of autocatalysis that is inconsistent with a JMAK-based formalization), or that the chemical environment is spatially and temporally invariant throughout the transformation. In contrast, equation (2.16) suggests that the chemical environment surrounding new nuclei differs from the chemical environment surrounding each of the already established and growing transformed regions. This description in turn suggests that new nuclei form far from the surface of existing grains (and the transformation involves a diffusion-limited compositional change). Real transformations might be presumed to fall somewhere between the conditions described by equations (2.3) and (2.16). One task of the present paper is to explore whether the predictions of the corresponding reaction equations are significantly different.

Replacing equation (2.3) with (2.16) in the derivation of the reaction equation from scenario 1, we obtain


(2.17) $$f\left(t\right)=1-\exp\left\{-\pi N_{0}G_{0}^{3}\left[\frac{1}{3}t^{4}+\frac{7\alpha +\beta }{15}t^{5}+\frac{9\alpha^{2}+4\alpha \beta }{45}t^{6}+\frac{6\alpha^{3}+7\alpha^{2}\beta }{210}t^{7}+\frac{\alpha^{3}\beta }{240}t^{8}\right]\right\}$$

for cases where growth rate and nucleation rate vary linearly with time.

The derivation follows the same general procedure as was used for the previous scenarios, and is provided in detail as electronic supplementary material, S4.

As a convenient check for consistency, we can set α=β=0, whereupon we again obtain the classic Avrami equation (2.5).

### 2.5. Scenario 5: derivation of an improved reaction equation, combining the improvements from scenarios 2 and 4

Here, the growth rate equation (2.1) and nucleation rate equation (2.2) in scenario 1 are, respectively, replaced with equations (2.6) and (2.7) from scenario 2, and equation (2.3) is replaced with equation (2.16) from scenario 4. These substitutions combine the separation of coefficients achieved in scenario 2 and the modified integration limits introduced in scenario 4.

The reaction equation is then


(2.18) $$f\left(t\right)=1-\exp\left\{-\pi \left[\begin{array}{c} \frac{N_{0}G_{0}^{3}}{3}t^{4}+\frac{7N_{0}G_{0}^{2}\alpha +G_{0}^{3}\beta }{15}t^{5}+\frac{9N_{0}G_{0}\alpha^{2}+4G_{0}^{2}\alpha \beta }{45}t^{6}\\ +\frac{6N_{0}\alpha^{3}+7G_{0}\alpha^{2}\beta }{210}t^{7}+\;\frac{\alpha^{3}\beta }{240}t^{8} \end{array}\right]\right\}$$

for cases where growth rate and nucleation rate vary linearly with time.

The derivation follows the same general procedure as was used for the previous scenarios, and is provided in detail as electronic supplementary material, S5.

Once again, if we set α=β=0, we obtain the classic Avrami equation (2.5).

### 2.6. Scenario 6: derivation of an improved reaction equation, combining the generalization from scenario 3 and the improvement from scenario 4

Here, the growth rate equation (2.1) and nucleation rate equation (2.2) in scenario 1 are, respectively, replaced with equations (2.9) and (2.10) from scenario 3, and equation (2.3) is replaced with equation (2.16) from scenario 4. These substitutions combine the generalized time-dependent growth rate and nucleation rate equations introduced in scenario 3 and the modified integration limits introduced in scenario 4.

The reaction equation is then


(2.19) $$f\left(t\right)=1-\exp\left\{-4\pi \;\left[\begin{array}{c} \frac{N_{0}G_{0}^{3}}{12}t^{4}+\frac{N_{0}G_{0}^{2}\alpha \left(p^{2}+4p+9\right)}{3\left(p+1\right)\left(p+3\right)\left(p+4\right)}t^{p+4}\\ +\frac{N_{0}G_{0}\alpha^{2}\left(4p^{2}+11p+9\right)}{{\left(p+1\right)}^{2}\left(p+3\right)\left(2p+3\right)\left(2p+4\right)}t^{2p+4}\\ +\frac{N_{0}\alpha^{3}\;}{{\left(p+1\right)}^{2}\left(2p+3\right)\left(3p+4\right)}t^{3p+4}\\ +\frac{G_{0}^{3}\beta }{60}t^{5}+\frac{G_{0}^{2}\alpha \beta \left(p^{3}+8p^{2}+19p+36\right)}{12\left(p+1\right)\left(p+3\right)\left(p+4\right)\left(p+5\right)}t^{p+5}\\ +\frac{G_{0}\alpha^{2}\beta \left(8p^{3}+37p^{2}+59p+36\right)}{{\left(p+1\right)}^{2\;}\left(p+3\right)\left(p+4\right)\left(2p+3\right)\left(2p+4\right)\left(2p+5\right)}t^{2p+5}\\ +\frac{\alpha^{3}\beta }{{\left(p+1\right)}^{2}\left(2p+3\right)\left(2p+4\right)\left(3p+5\right)}t^{3p+5} \end{array}\right]\right\}.$$

The derivation follows the same general procedure as was used for the previous scenarios, and is provided in detail as electronic supplementary material, S6. In this case, one of the integrations does not yield an analytic solution for all combinations of the parameters *p* and *q*. Equation (2.19) was obtained by keeping p unrestricted while assuming the simplest (i.e. linear) variation of nucleation rate with time, setting q=1. Therefore, q does not appear in equation (2.19).

If we additionally set p=1, equation (2.19) reduces to the final reaction equation from scenario 5, as expected. Another reality check can be performed on the reaction equation (2.19) by setting $G_{0}=0$ , which reduces the equation to


(2.20) $$f\left(t\right)=1-\exp\left\{-4\pi \;\left[\begin{array}{c} \frac{N_{0}\alpha^{3}\;}{{\left(p+1\right)}^{2}\left(2p+3\right)\left(3p+4\right)}t^{3p+4}\\ +\frac{\alpha^{3}\beta }{{\left(p+1\right)}^{2\;}\left(2p+3\right)\left(2p+4\right)\left(3p+5\right)}t^{3p+5} \end{array}\right]\right\}.$$

Consistent with scenario 3, equation (2.20) will apply to all cases of growth that follow equation (2.12), including those governed by case I (Fickian) diffusion kinetics, case II diffusion (interface control), anomalous diffusion and super case II diffusion—with the constraint that we needed q=1 in order to derive equation (2.19) and therefore equation (2.20). If we now consider a transformation that is specifically governed by case II diffusion (interface control), such as the condensation or crystallization of a pure substance, or the recrystallization of a pure solid, then growth rate is constant and p=0. In addition, let β=0, so that nucleation rate becomes constant. Equation (2.20) reduces to the classic Avrami equation for sporadic nucleation and three-dimensional growth at a constant rate (2.5), as expected, with the role of initial growth rate represented here by α.

We noted in connection with deriving equation (2.19) that one of the integrations does not yield an analytic solution for all combinations of the parameters *p* and *q*. We chose to keep p unrestricted while setting q=1. However, there are many other situations that we could have explored. For another straightforward example, we can start by setting $G_{0}=0$ and p=−0.5, in which case, the growth rate equation that feeds into the derivation of the reaction equation is $G\left(t\right)=G_{0}+\alpha t^{p}=\alpha t^{-0.5}$, indicating growth that is dominated by classical Fickian diffusion; we will allow q to remain unrestricted. The reaction equation is then


(2.21) $$f\left(t\right)=1-\exp\left\{-16\pi \;\alpha^{3}\left[\frac{N_{0}}{5}t^{2.5}+\frac{\beta }{\left(q+1\right)\left(q+2\right)\left(q+2.5\right)}t^{q+2.5}\right]\right\}.$$

The derivation follows the same general procedure as was used for deriving equation (2.19), and is provided in detail as electronic supplementary material, S7.

## 3. Focus on the time dependence of nucleation rate

In scenarios 3 and 6, we focused particular attention on the time dependence of growth in the context of case I (Fickian) diffusion, case II diffusion (interface control), anomalous diffusion and super case II diffusion. We will now consider the time dependence of nucleation in more detail for these contexts.

### 3.1. Time dependence of nucleation rate in scenario 3

Setting $G_{0}=0$ (required for the contexts under consideration, as noted in §2.3) and q=1 (linear variation of nucleation rate with time) reduces the general reaction equation (2.11) for scenario 3 to


(3.1) $$f\left(t\right)=1-\exp\left\{-4\pi \alpha^{3}\left[\begin{array}{c} \frac{2N_{0}\;}{\left(p+2\right)\left(2p+3\right)\left(3p+4\right)}t^{3p+4}\\ +\frac{\beta \;}{\left(p+3\right)\left(2p+4\right)\left(3p+5\right)}t^{3p+5} \end{array}\right]\right\}$$

with the role of initial growth rate being fulfilled by α.

If the initial nucleation rate is small and then increases linearly, we can set $N_{0}∼0$ in equation (3.1). The reaction equation then reduces further to


(3.2) $$f\left(t\right)=1-\exp\left[-\frac{4\pi \alpha^{3}\beta \;}{\left(p+3\right)\left(2p+4\right)\left(3p+5\right)}t^{3p+5}\right].$$

We are not aware of a material transformation that exhibits this type of kinetic behaviour. However, the possible applications of the Avrami equation in other fields, such as epidemiology [15,25], provides a background against which such behaviour becomes relevant. For example, the COVID-19 pandemic originated at a single locality, but new nuclei then appeared rapidly in places far from that origin as the virus spread via human travel.

For a constant sporadic nucleation rate, $N_{0}$ is finite and β=0. In this case equation (3.1) reduces to


(3.3) $$f\left(t\right)=1-\exp\left[-\frac{8\pi N_{0}\alpha^{3}}{\left(p+2\right)\left(2p+3\right)\left(3p+4\right)}t^{3p+4}\right].$$

The reaction equation for instantaneous nucleation cannot be obtained from equation (3.1), because the latter was derived via a process that assigned nucleation rate as a continuous function of time. For instantaneous nucleation, all the nucleation activity is concentrated at t=0, and we have to modify the derivation of the reaction equation accordingly, with the number of nuclei per unit volume remaining at its initial value $N_{0,i}$ throughout the transformation. The reaction equation for scenario 3 with this modification is


(3.4) $$f\left(t\right)=1-\exp\left[-\frac{4\pi N_{0,i}\alpha^{3}}{{\left(p+1\right)}^{2}\;\left(3p+3\right)}t^{3p+3}\right]$$

with the detailed derivation provided as electronic supplementary material, S8.

For three-dimensional interface-controlled growth (p=0), we note that the Avrami exponent (the power to which t is raised in these equations) would be equal to 5, 4 and 3, respectively, for a linearly increasing nucleation rate, a constant sporadic nucleation rate and instantaneous nucleation. The exponents for sporadic and instantaneous nucleation rates are well documented in materials science textbooks. However, in a context where the Avrami equation might be applied to transformations that involve a linearly increasing nucleation rate, and interface-controlled growth, an Avrami exponent of 5 is possible. Indeed, even higher values of the exponent could be realized if the nucleation rate increases more steeply with time (q>1). For example, we can consider setting $G_{0}=0$ and p=0 (interface control) in the general reaction equation (2.11) for scenario 3, leading to


(3.5) $$f\left(t\right)=1-\exp\left\{-\pi \left[\frac{N_{0}\alpha^{3}\;}{3}t^{4}+\frac{8\alpha^{3}\beta \;}{\left(q+1\right)\left(q+2\right)\left(q+3\right)\left(q+4\right)}t^{q+4}\right]\right\}.$$

If the initial nucleation rate is small and then increases according to $t^{q}$, we can set $N_{0}∼0$ in equation (3.5), leaving us with


(3.6) $$f\left(t\right)=1-\exp\left[-\frac{8\pi \alpha^{3}\beta \;}{\left(q+1\right)\left(q+2\right)\left(q+3\right)\left(q+4\right)}t^{q+4}\right].$$

### 3.2. Time dependence of nucleation rate in scenario 6

Setting $G_{0}=0$ (required for the contexts under consideration, as noted in §2.3) reduces the general reaction equation (2.19) for scenario 6 to


(3.7) $$f\left(t\right)=1-\exp\left\{-\frac{4\pi \alpha^{3}}{{\left(p+1\right)}^{2}}\;\left[\frac{N_{0}\;}{\left(2p+3\right)\left(3p+4\right)}t^{3p+4}+\frac{\beta }{\left(2p+3\right)\left(2p+4\right)\left(3p+5\right)}t^{3p+5}\right]\right\}$$

with the role of initial growth rate being fulfilled by α. (Recall that this equation does not contain q, because it was necessary to set q=1 in the derivation of equation (2.19) in order to obtain an analytic solution to an integral in that derivation.)

If the initial nucleation rate is small and then increases linearly, we can set $N_{0}∼0$ in equation (3.7). The reaction equation then reduces further to


(3.8) $$f\left(t\right)=1-\exp\left[-\frac{4\pi \alpha^{3}\beta \;}{{\left(p+1\right)}^{2}\left(2p+3\right)\left(2p+4\right)\left(3p+5\right)}t^{3p+5}\right].$$

For a constant sporadic nucleation rate, $N_{0}$ is finite and β=0. In this case equation (3.7) reduces to


(3.9) $$f\left(t\right)=1-\exp\left[-\frac{4\pi N_{0}\alpha^{3}}{{\left(p+1\right)}^{2}\left(2p+3\right)\left(3p+4\right)}t^{3p+4}\right].$$

For instantaneous nucleation, we have to modify the derivation of the reaction equation to account for the number of nuclei per unit volume remaining at its initial value $N_{0,i}$ throughout the transformation, leading to


(3.10) $$f\left(t\right)=1-\exp\left[-\frac{4\pi N_{0,i}\alpha^{3}}{{\left(p+1\right)}^{2}\left(3p+3\right)}t^{3p+3}\right].$$

The derivation is similar to the one that was used to obtain equation (3.4) and is provided as electronic supplementary material, S9. Equation (3.10) is identical to equation (3.4). This is due to the integration limits of growth rate becoming the same for scenarios 3 and 6 when nucleation is instantaneous.

The discussion in §3.1, regarding the time dependence of nucleation rate in scenario 3, is also relevant here. An Avrami exponent of 5 is possible in a context that involves a linearly increasing nucleation rate and interface-controlled growth. If we revisit the derivation of the scenario 6 reaction equation, setting $G_{0}=0$ and p=0 (interface control) and allowing *q* to remain unrestricted, we obtain the analytic solution


(3.11) $$f\left(t\right)=1-\exp\left\{-\pi \left[\frac{N_{0}\alpha^{3}\;}{3}t^{4}+\frac{8\alpha^{3}\beta \;}{\left(q+1\right)\left(q+2\right)\left(q+3\right)\left(q+4\right)}t^{q+4}\right]\right\}$$

via the detailed derivation provided in electronic supplementary material, S10. Equation (3.11) is identical to equation (3.5), which was obtained for these conditions in scenario 3. This is because the integrand for growth reduces to a constant (α) when $G_{0}=0$ and p=0, and therefore the different integration limits of scenarios 3 and 6 yield the same results.

If the initial nucleation rate is small and then increases according to $t^{q}$, we can set $N_{0}∼0$ in equation (3.11), leaving us with


(3.12) $$f\left(t\right)=1-\exp\left[-\frac{8\pi \alpha^{3}\beta \;}{\left(q+1\right)\left(q+2\right)\left(q+3\right)\left(q+4\right)}t^{q+4}\right]$$

and again demonstrating that the time dependence of nucleation rate can lead to an Avrami exponent that is greater than 4. Note that changing the time dependence of nucleation is not just reflected in the Avrami exponent n, but also in the value of the numerical factor embedded in k. Similarly, the kinetics of growth also affect k as well as n. Table 2 summarizes the equations for calculating the Avrami coefficient k for different time dependences of nucleation and two common growth modes, derived for scenarios 3 and 6.

**Table 2.** Equations describing the Avrami coefficient *k* for different time dependences of nucleation in scenarios 3 and 6, for interface-controlled and diffusion-controlled growth.

nucleation type	scenario and equation	growth under interface control (p=0)	growth under diffusional control (p=-1/2)
instantaneous	3 (3.4)	$k=\frac{4\pi N_{0,i}\alpha^{3}}{3}$	$k=\frac{32\pi N_{0,i}\alpha^{3}}{3}$
	6 (3.10)	$k=\frac{4\pi N_{0,i}\alpha^{3}}{3}$	$k=\frac{32\pi N_{0,i}\alpha^{3}}{3}$
sporadic; constant rate	3 (3.3)	$k=\frac{{\pi N}_{0}\alpha^{3}}{3}$	$k=\frac{16{\pi N}_{0}\alpha^{3}}{15}$
	6 (3.9)	$k=\frac{{\pi N}_{0}\alpha^{3}}{3}$	$k=\frac{16{\pi N}_{0}\alpha^{3}}{5}$
linear rate increase; initial rate is small	3 (3.2)	$k=\frac{\pi \alpha^{3}\beta }{15}$	$k=\frac{16\pi \alpha^{3}\beta }{105}$
	6 (3.8)	$k=\frac{\pi \alpha^{3}\beta }{15}$	$k=\frac{16\pi \alpha^{3}\beta }{21}$

## 4. Exploring some predictions of scenarios 1 through 6

### 4.1. Obtaining values of the coefficients α and β for linear time dependence of growth rate and nucleation rate

For scenarios 1, 2, 4 and 5, we need a procedure whereby meaningful values of α and β can be chosen so that the final growth rate and nucleation rate are a desired percentage (or multiple, or fraction) of the initial growth rate and nucleation rate, respectively. Johnson & Mehl [7] employ such a procedure for scenario 1, but do not provide the equations that they used.

The method involves using the time needed for 99.9% transformation as a reference, and calculating either the value of α needed to increase or decrease the growth rate by a factor x (while nucleation rate is constant, i.e. β is zero), or the value of β needed to increase or decrease the nucleation rate by a factor y (while growth rate is constant, i.e. α is zero), during that time. Plots of the reaction equation for different values of α (for a given initial growth rate, and nucleation rate constant), or for different values of β (for a given initial nucleation rate, and growth rate constant) can be constructed to illustrate how separately changing α or β affects the transformation.

For scenario 1, with changing growth rate and constant nucleation rate (β=0), we obtain


(4.1) $$\alpha_{1}=\left(x-1\right){\left[\;\frac{\pi N_{0}G_{0}^{3}\;\left(\frac{1}{42}+\frac{2}{21}x+\frac{29}{210}x^{2}+\frac{8}{105}x^{3}\right)}{-\ln0.001}\right]}^{1/4}.$$

For scenario 1, with changing nucleation rate and constant growth rate (α=0) we obtain


(4.2) $$\;\beta_{1}=\left(y-1\right){\left[\;\frac{\frac{\pi N_{0}G_{0}^{3}}{15}\;\left(4+y\right)}{-\ln0.001}\right]}^{1/4}.$$

The subscript attached to the parameters α and β denotes the scenario for which parameters are calculated. Only the positive roots have physical significance in equations (4.1) and (4.2). The detailed derivation of these equations is provided in electronic supplementary material, S11. The corresponding equations for scenario 4 are derived by the same process and are also shown in electronic supplementary material, S11.

We do not derive the corresponding equations for scenarios 2 and 5, because these scenarios respectively predict the same behavior as scenarios 1 and 4. The equivalence arises because of simple relationships between parameters: $\alpha_{2}={G_{0}\alpha }_{1}$ , $\beta_{2}=N_{0}\beta_{1}$ , $\alpha_{5}=G_{0}\alpha_{4}$ and $\beta_{5}=N_{0}\beta_{4}$.

Table 3 reports the values for α and β calculated by following the procedure described above, and using the illustrative initial and final values of growth rate and nucleation rate that were adopted by Johnson & Mehl [7] ($G_{0}=10^{-5}\;cm\;s^{-1}$ and $N_{0}=10^{3}\;cm^{-3}\;s^{-1}$).

**Table 3.** Values of *α* and *β* in scenarios with linear time dependencies of nucleation rate and growth rate. Units of α are $s^{-1}$ for scenarios 1 and 4, and $cm\;s^{-2}$ for scenarios 2 and 5. Units of β are $s^{-1}$ for scenarios 1 and 4, and $cm^{-3}\;s^{-2}$ for scenarios 2 and 5.

condition	x	y	S1	S2	S4	S5
decreased G constant N	0.33	1	$\alpha_{1}=$ −2.86 × 10^−4^	$\alpha_{2}=$−2.86 × 10^−9^	$\alpha_{4}=$−3.11 × 10^−4^	$\alpha_{5}=$−3.11 × 10^−9^
increased G constant N	3	1	$\alpha_{1}=$ 2.26 × 10^−3^	$\alpha_{2}=$ 2.26 × 10^−8^	$\alpha_{4}=$ 2.02 × 10^−3^	$\alpha_{5}=$ 2.02 × 10^−8^
decreased N constant G	1	0	$\beta_{1}=$ −5.90 × 10^−4^	$\beta_{2}=$ −5.90 × 10^−1^	$\beta_{4}=$ −5.90 × 10^−4^	$\beta_{5}=$ −5.90 × 10^−1^
increased N constant G	1	10	$\beta_{1}=$ 7.26 × 10^−3^	$\beta_{2}=$ 7.26 × 10^0^	$\beta_{4}=$ 7.26 × 10^−3^	$\beta_{5}=$ 7.26 × 10^0^

We must be careful to check whether a negative value of α or β has resulted in a physically impossible negative value for growth rate or nucleation rate before the reaction reached 99.9% completion.

**Figure 2.**
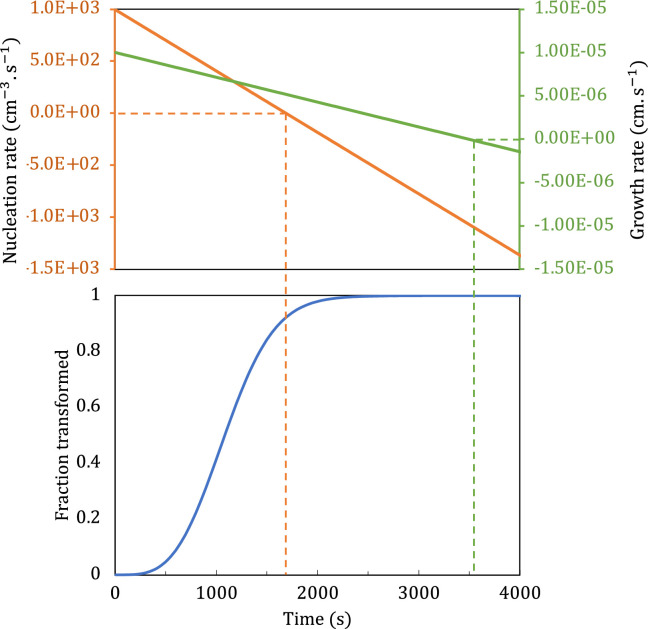
(Top) Nucleation rate (red; left-hand ordinate) and growth rate (green; right-hand ordinate) as functions of time when $\alpha_{2}=-2.86\times 10^{-9}\;cm\;s^{-2}$, $\beta_{2}=-5.90\times 10^{-1}\;cm^{-3}\;s^{-2}$, $G_{0}=1\times 10^{-5}\;cm\;s^{-1}$, and $N_{0}=1\times 10^{3}cm^{-3}\;s^{-1}$. (Bottom) Plot of corresponding transformation curve obtained with reaction equation (2.8).

Figure 2 shows an example of how such contradictory behaviour arises if we simultaneously insert the negative values of α and β from table 3 into the reaction equation from scenario 2. In this example, it is specifically the nucleation rate that becomes negative before the reaction completes.

The results described below do not contain any such unphysical situations.

### 4.2. Comparing responses of scenarios 2 and 5 reaction equations to changes in α andβ

In this section, we explore how the progress of phase transformation, as predicted by the reaction equations, is affected by increasing or decreasing either the nucleation rate or the growth rate linearly with time.

**Figure 3.**
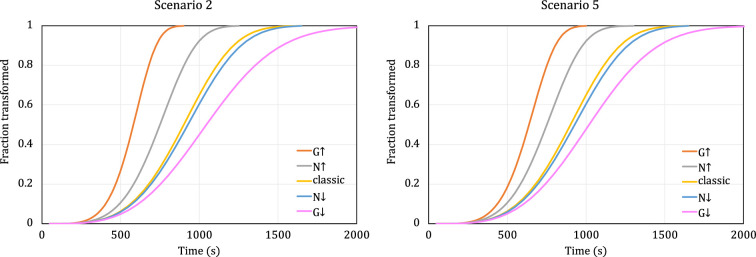
Transformation curves modelled by scenario 2 (left) and scenario 5 (right) with linear increase (upward arrow) or decrease (downward arrow) in either growth rate or nucleation rate. The initial rates for all curves are $G_{0}=10^{-5}\;cm\;s^{-1}$ and $N_{0}=10^{3}\;cm^{-3}\;s^{-1}$. The yellow (middle) curve shows the prediction of the classic JMAK model with constant rates of nucleation and growth. Note that scenarios 1 and 4 would yield the same plots as scenarios 2 and 5, respectively.

Figure 3 shows plots of phase transformations starting at a growth rate of $G_{0}=10^{-5}\;cm\;s^{-1}$ and a nucleation rate of $N_{0}=10^{3}\;cm^{-3}\;s^{-1}$, modelled by scenario 2 (equation (2.8)) and scenario 5 (equation (2.18)). These initial values of $G_{0}$ and $N_{0}$ are the same as those used by Johnson & Mehl [7]. The values of α and β also correspond to those used by Johnson & Mehl, scaled to match the refinements made in our scenarios 2 and 5, as recorded in table 3. As shown in the figure, corresponding transformation curves are identical for both scenarios in the cases where growth rate is constant; this is expected, because these two scenarios only differ in the integration of growth.

For constant nucleation rate and decreasing growth rate (negative α), figure 3 shows that scenario 5 reaches completion faster than scenario 2. In contrast, for constant nucleation rate and increasing growth rate (positive α), scenario 2 reaches completion faster than scenario 5. As noted in §2.4, where we introduced the rationale for modifying Johnson & Mehl’s integration limits when calculating growth, the behaviour of a real transformation can be presumed to fall somewhere between the extremes predicted by scenario 2 (or scenario 1) and scenario 5 (or scenario 4).

### 4.3. Comparing predictions of scenarios 3 and 6 reaction equations for two common growth types

In this section, we explore how the progress of phase transformation is predicted by the scenarios 3 and 6 reaction equations in two widely encountered practical situations. We focus on the two growth types that are most commonly discussed in materials science textbooks [17,18] in the context of JMAK kinetics: parabolic growth (p=-0.5), and interface-controlled growth (p=0). For both cases, we set $G_{0}=0$ (so that the initial growth rate is represented by α) and $N_{0}=10^{5}\;cm^{-3}\;s^{-1}$.

**Figure 4.**
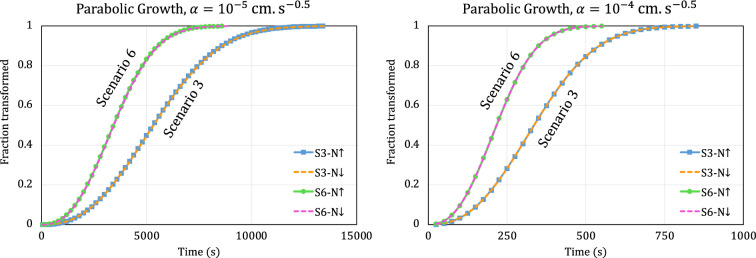
Transformation curves modelled by scenarios 3 and 6 with parabolic growth for $\alpha =10^{-5}\;cm\;s^{-0.5}$ (left) and $\alpha =10^{-4}\;cm\;s^{-0.5}$ (right) and linearly increasing (upward arrow) or linearly decreasing (downward arrow) nucleation rate.

For parabolic growth (where growth rate is an intrinsically decreasing nonlinear function of time), we explore the effect of linearly increasing and decreasing the nucleation rate by using $\beta =0.5\;cm^{-3}\;s^{-2}$ and $\beta =-0.5\;cm^{-3}\;s^{-2}$, respectively, and keeping q=1. The results for $\alpha =10^{-5}\;cm\;s^{-0.5}$ and $\alpha =10^{-4}\;cm\;s^{-0.5}$ are shown in figure 4.

Comparison of figures 3 and 4 demonstrates that the sensitivity of the phase transformation to a particular change in β is readily apparent when the growth rate is constant, but negligible when growth is parabolic.

**Figure 5.**
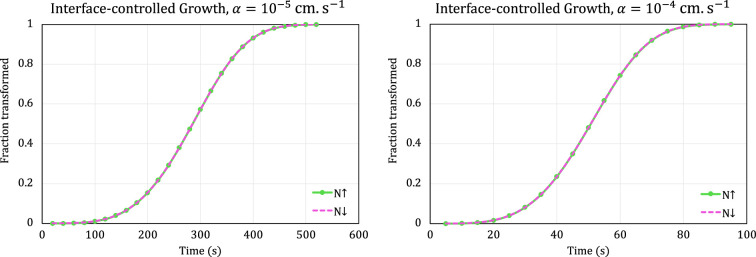
Transformation curves modelled by scenarios 3 and 6 for interface-controlled growth with $\alpha =10^{-5}\;cm\;s^{-1}$ (left) and $\alpha =10^{-4}\;cm\;s^{-1}$ (right) and linearly increasing (upward arrow) or linearly decreasing (downward arrow) nucleation rate. For interface-controlled growth together with linear nucleation rate, the reaction equations of scenarios 3 and 6 are identical.

For interface-controlled growth, having p=0 automatically keeps the growth rate constant, and we explore the effect of increasing and decreasing the nucleation rate. We again use $\beta =0.5\;cm^{-3}\;s^{-2}$ and $\beta =-0.5\;cm^{-3}\;s^{-2}$, and we keep q=1. The results for $\alpha =10^{-5}\;cm\;s^{-1}$ and $\alpha =10^{-4}\;cm\;s^{-1}$ are shown in figure 5.

Comparison of figures 4 and 5 clearly illustrates that, as expected, for the same values of initial growth rate and time-dependent nucleation rate, the interface-controlled process will proceed much more quickly than the diffusion-controlled process.

### 4.4. Comparing predictions of scenarios 3 and 6 reaction equations for two common nucleation types

We now explore how the progress of phase transformation is predicted by the scenarios 3 and 6 reaction equations under two widely encountered nucleation conditions: sporadic nucleation at a constant rate, and instantaneous nucleation.

**Figure 6.**
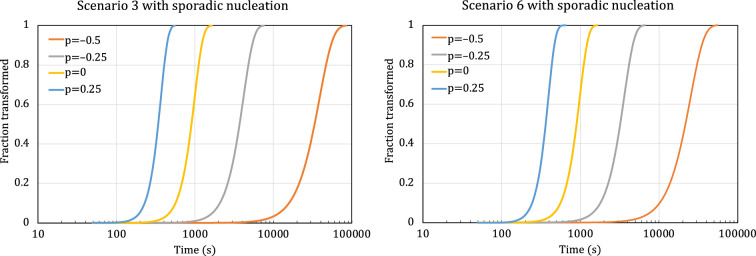
Plots of phase transformations with sporadic nucleation at constant rate of $N_{0}=10^{3}\;cm^{-3}\;s^{-1}$ modelled by scenario 3 (left) and scenario 6 (right) for standard growth types.

Figure 6 shows the progress of phase transformation with constant sporadic nucleation rate, as predicted by the scenarios 3 and 6 reaction equations, for standard diffusion processes that were described in table 1. The nucleation rate is $N_{0}=10^{3}\;cm^{-3}\;s^{-1}$, and the role of initial growth rate is fulfilled by $\alpha =10^{-5}$ (units differ based on the growth type).

**Figure 7.**
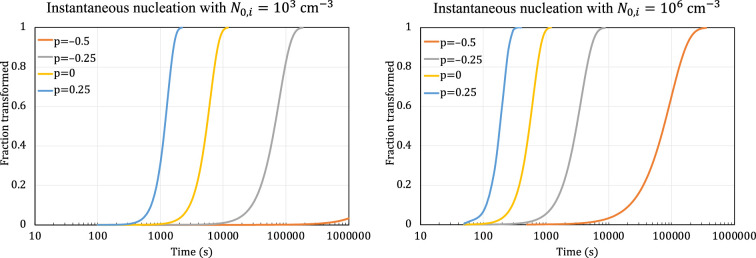
Plots of phase transformations with instantaneous nucleation modelled by scenarios 3 and 6 for standard growth types. The number of initial nuclei is $N_{0,i}=10^{3}\;cm^{-3}$ (left), and $N_{0,i}=10^{6}\;cm^{-3}$ (right). For instantaneous nucleation, the reaction equations of scenarios 3 and 6 are identical. The initial growth rate is $10^{-5}$ (units differ based on the growth type).

Figure 7 shows the progress of phase transformation with instantaneous nucleation, as predicted by the scenarios 3 and 6 reaction equations for the standard diffusion processes that were described in table 1. The number of initial nuclei is $N_{0,i}=10^{3}\;cm^{-3}$, and the role of initial growth rate is fulfilled by $\alpha =10^{-5}$ (units differ based on the growth type).

The plots in figures 6 and 7 demonstrate the impact of the different diffusion types on the overall shape of the transformation curves, highlighting the significance of our ability to accommodate these different diffusion types in our generalized derivation of the reaction equation. The results are also qualitatively consistent with simple mechanistic understanding of the common nucleation types and growth types, and therefore provide some validation of the equations.

## 5. Conclusions

— The JMAK equation, commonly known as the Avrami equation, is widely employed for modelling the fraction of a system that has been transformed as a function of time during processes that are analogous to nucleation-and-growth-type phase transformations. Its convenience as a modelling tool within and beyond materials chemistry reflects its relative simplicity and light computational burden.
— The algebraic representation of growth rate G(t) and nucleation rate N(t) that vary linearly with time in Johnson & Mehl’s derivation of a classic reaction equation for phase transformations [7,20] can be re-stated in a more useful format.
— A case can be made for modifying the integration limits that Johnson & Mehl use for calculating growth in the derivation of their reaction equation.
— The formulation of G(t) and N(t) can be easily extended and generalized to obtain reaction equations for modelling practical contexts that include case I (Fickian) diffusion, case II diffusion (interface control), anomalous diffusion, and super case II diffusion.
— The generalized formulation of G(t) and N(t) reveals the possibility of Avrami exponents that exceed the classical maximum value of 4 (obtained when growth occurs at a constant rate in three dimensions, and nucleation occurs at constant sporadic rate). A value of 5 is predicted for the combination of three-dimensional growth at a constant rate and a linearly increasing nucleation rate.
— Isothermal phase transformations involving a nucleation rate that increases linearly with time would be exceptional in materials chemistry, but an analogous model is potentially relevant to gaining insight about some non-thermodynamic transformations. One example can be the pandemic spread of a virus where new infection clusters appear in places removed from existing ones as the virus spreads via human travel.
— The reaction equations obtained from the generalized formulation of G(t) and N(t) show how the time dependence of nucleation rate and growth rate affect not only the Avrami exponent n but also the Avrami coefficient k.
— The derivation of the reaction equations retains the mathematical accessibility of Johnson & Mehl’s original paper.

## Data Availability

This article has no additional data. All derived equations presented in the main text are substantiated in the accompanying electronic supplementary material [26].
